# Supplementary material for: Twofold symmetry of c-axis resistivity in topological kagome superconductor CsV3Sb5 with in-plane rotating magnetic field
Source: Nat Commun. 2021 Nov 18;12:6727. doi: 10.1038/s41467-021-27084-z (PMC8602318; doi:10.1038/s41467-021-27084-z)
Supplement: Supplementary file 1 — Supplementary Information [file 41467_2021_27084_MOESM1_ESM.pdf]

Supplementary Information

Twofold symmetry of *c*-axis resistivity in topological kagome superconductor CsV_3Sb_5 with in-plane rotating magnetic field

Ying Xiang[1†], Qing Li[1†], Yongkai Li[2,3†], Wei Xie[1], Huan Yang[1*], Zhiwei Wang[2,3*], Yugui Yao[2,3], and Hai-Hu Wen[1*]

[1]National Laboratory of Solid State Microstructures and Department of Physics, Collaborative Innovation Center of Advanced Microstructures, Nanjing University, Nanjing 210093, China

[2]Key Laboratory of Advanced Optoelectronic Quantum Architecture and Measurement, Ministry of Education, School of Physics, Beijing Institute of Technology, Beijing 100081, China

[3]Micronano Center, Beijing Key Lab of Nanophotonics and Ultrafine Optoelectronic Systems, Beijing Institute of Technology, Beijing 100081, China

Supplementary Note 1: Structure and stoichiometry of CsV_3Sb_5 single crystals.

The crystal structure of CsV_3Sb_5 was analyzed via X-ray diffraction (XRD) by using a θ-2θ scan. A typical XRD pattern is shown in Supplementary Fig. 1, and one can see sharp (00*l*) reflection peaks. The full width at half maximum of the (004) peak is only 0.06°, which indicates the high quality of the single crystal. The stoichiometry of the single crystal is characterized by the energy dispersive spectrum (EDS) measurement. Supplementary Fig. 2 shows a typical EDS spectrum, and the compositions of Cs : V : Sb = 1.14 : 2.84 : 5 which is near stoichiometric compositions of 1 : 3 : 5. The slightly excess of Cs and Sb may be due to the self-flux method in the sample growth procedure with Cs-Sb binary eutectic mixture as the flux or the error in the determination of the magnetic element V. Based on scanning tunneling microscopy data carried out in the same batch of samples[1], we always observe perfect atomically resolved topographies in STM measurements, and we have not observed the vanadium vacancies.

Supplementary Note 2: Determination of in-plane crystallographic axes of single crystals from Laue diffraction patterns.

Owing to the quasi-two-dimensional crystal structure of CsV_3Sb_5, the *c*-axis direction is very easy to determine in these flake-shaped sample. There are some naturally formed edges with the angle of about 120º for neighboured edges (Supplementary Fig. 3). The in-plane axis direction of crystal cannot be confirmed only by the shape of the sample, because there are two kinds of axis direction in the hexagonal structure: vertex direction (*a*', indicated by a blue arrow in Supplementary

Fig. 4b) and edge direction (*a*, indicated by a red arrow in Supplementary Fig. 4b). In order to determine the in-plane crystalline axis directions of crystal, one straight way is to perform the in-plane Laue diffraction experiment. However, the sample is too thin, and such an experiment is very difficult to do. Therefore, we try to distinguish the in-plane crystalline axis directions of crystal through the Laue diffraction pattern measured along the *c*-axis. The obtained Laue diffraction pattern (Supplementary Fig. 4c) shows two sets of six-fold symmetry axis, and the directions are the same as two sets of symmetry-axis directions based on the sample shape. Derived from the experimental configuration of Laue diffraction measurements, the arrows along the same direction in Laue diffraction pattern and the sample are plotted by the same colour in Supplementary Fig. 4b,c. Then we try to fix the exact in-plane crystalline axes based on the simulated result (Supplementary Fig. 4d).

Supplementary Fig. 5 shows the comparison of characteristic spots from the experimental and theoretically simulated Laue diffraction patterns; these spots help us distinguish crystalline axes. In Supplementary Fig. 5a,b, some spots with strong intensity (marked by yellow circles) can be seen, which should be the typical spots along another set of axes (*a*’) instead of the in-plane crystalline axis direction. In addition, some special bowknot-like patterns, which are consisted by five spots marked in Supplementary Fig. 5c,d, further confirm the crystalline axis direction. Combining the above two evidences, we can conclude that *a*-direction instead of *a*’-direction is the direction of crystalline axis. These directions are just along directions of sample natural edges.

Supplementary Note 3: Analysis of $\rho_c(\theta)$ curves measured at high magnetic fields.

The $\rho_c(\theta)$ curves measured at high magnetic fields show clear oscillations with a six-fold symmetry besides the twofold one. Then we try to analyze the components of these signal from $\rho_c(\theta)$ curves measured in different samples at 2 K and under the magnetic field of 7 T. Cosine functions with different period including 180°, 90°, 60°, and 30° are used to fit $\rho_c(\theta)$ curves. Fitting results are shown in Supplementary Fig. 6. It should be noted that the fourfold symmetric component is also twofold symmetric, i.e., the fourfold symmetric component should have two mirror symmetrical axes. This means that the nematicity cannot be easily expressed as a cosine function with period of 180°, and the fourfold symmetric component is required to further refine the detail of the nematicity. Similarly, the 12-fold symmetric component is certainly six-fold symmetric. Therefore, we use the summation of components with twofold (with the period of 180°) and fourfold (with the period of 90°) symmetry as the contribution from the nematic electronic states, while we use the summation of the components with six-fold (with the period of 60°) and 12-fold (with the period of 30°) symmetry as the contribution from the electronic states due to the six-fold electronic structure in this material. The nematic electronic state with larger resistivity contribution is always along one pair of in-plane crystalline axes, while the signal with six-fold symmetry is misaligned from the *a*-axis determined from the crystal shape in sample 1. The relatively large misalignment may be from the larger thickness of sample 1, and there may be some distortion of different layers in this layered material.

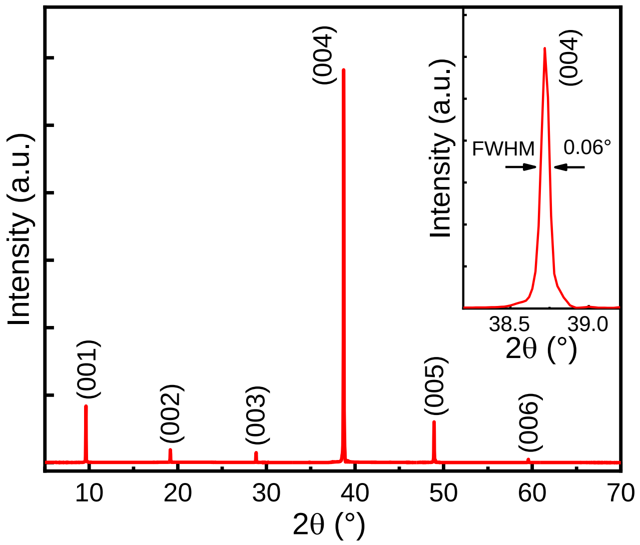

Supplementary Fig. 1 | A typical XRD pattern of a CsV_3Sb_5 single crystal. The inset shows an enlarged view of the (004) peak, and the full width at half maximum of 0.06° indicates very high quality of the single crystal.

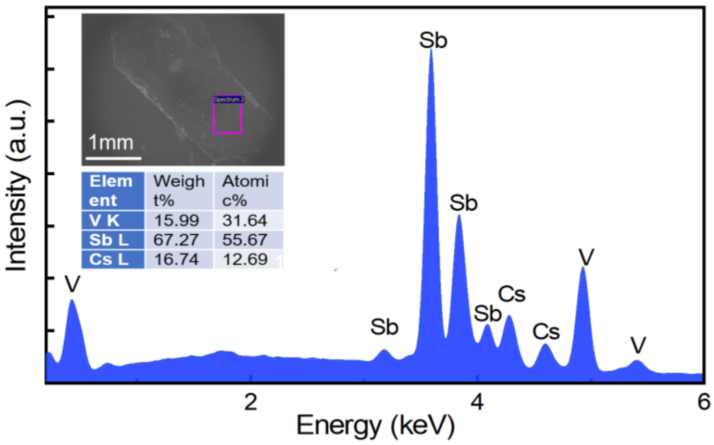

Supplementary Fig. 2 | A typical EDX spectrum of a CsV_3Sb_5 single crystal. The inset shows SEM image and the chemical composition of CsV_3Sb_5.

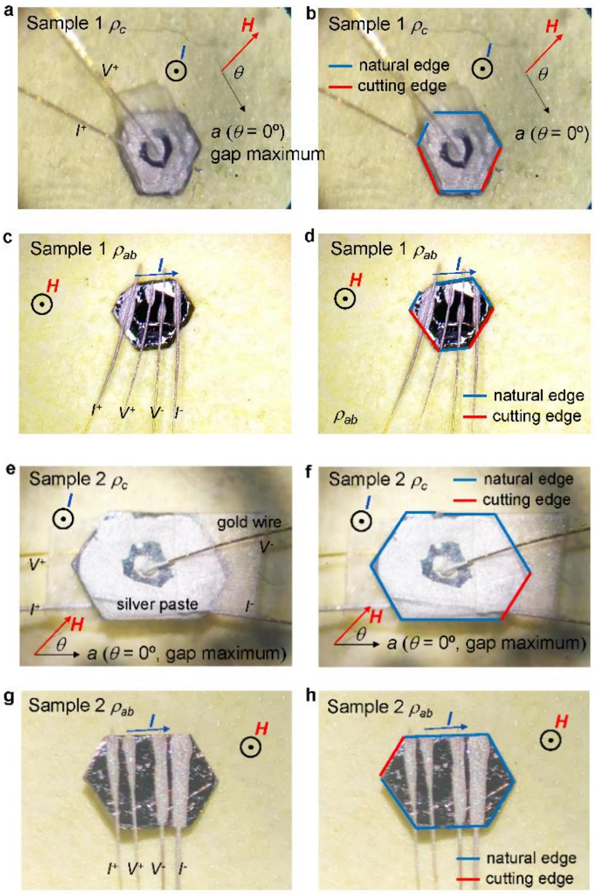

Supplementary Fig. 3 | Experimental configuration for resistivity measurements. Experimental configuration for (**a**,**b**,**e**,**f**) *c*-axis and (**c**,**d**,**g**,**h**) *ab*-plane resistivity measurement for (**a**-**d**) sample 1 and (**e**-**h**) sample 2. The experimental data shown in Figs. 1-4 were measured in sample 1, while the data shown in Fig. 5 were measured in sample 2. The naturally formed edges and cutting edges are marked by lines with different colours. The superconducting gap maximum direction is approximately near one pair of in-plane crystallographic axes which is marked as the *a*-axis in **a**,**b**,**e**,**f**. Sample 2 is bigger but thinner than sample 1, then the noise for the resistance measurement is much larger. The two samples are mounted on the sample holder with the relative angle relation as shown in **a** and **e**, but the directions of superconducting gap maximum have an intersect angle of about 60°.

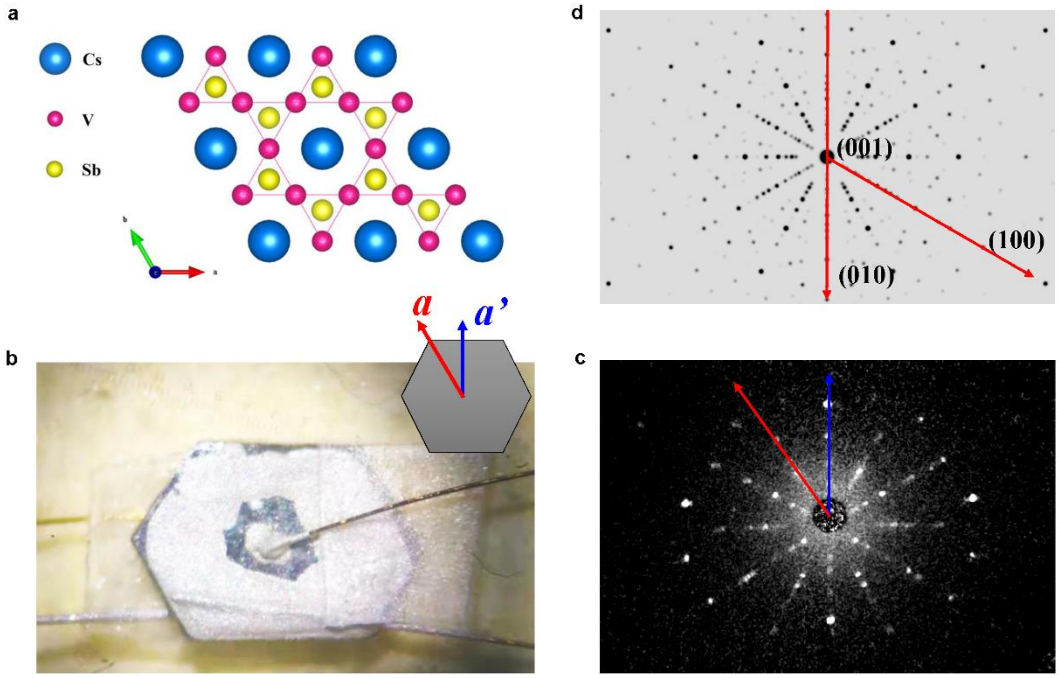

Supplementary Fig. 4 | Crystal structure, typical sample shape, and Laue diffraction data. a, Crystal structure of CsV_3Sb_5. **b**, Sample shape of a piece of single crystal. Inset in **b** shows two directions of symmetry axes according to the sample shape, and these two directions may be the candidate of the in-plane crystalline axis. **c**,**d**, Experimentally obtained and theoretically simulated Laue diffraction patterns along 001 direction of CsV_3Sb_5, respectively. The experimental image was taken with one edge of the crystal in the horizontal direction.

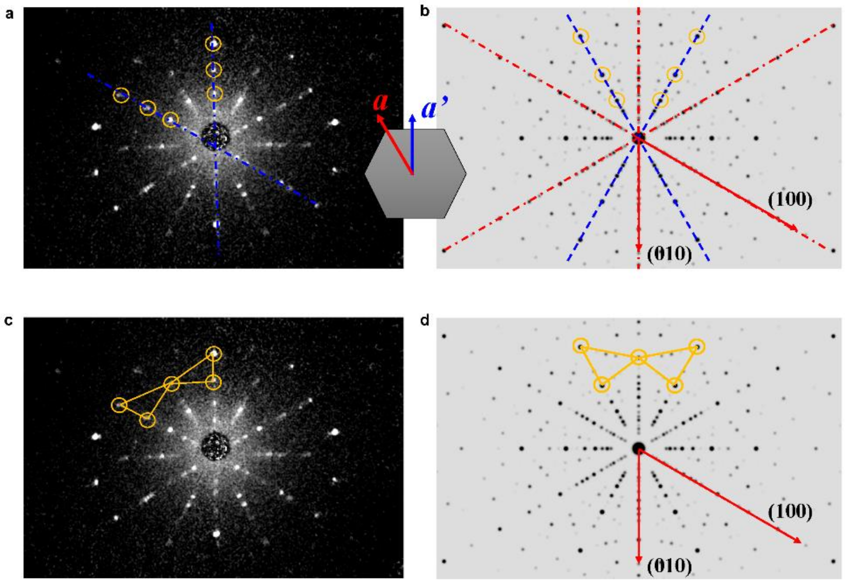

Supplementary Fig. 5 | Comparative study of experimental Laue diffraction pattern and theoretically simulated Laue diffraction pattern in CsV_3Sb_5. a,**c**, Experimentally obtained Laue diffraction pattern along 001 direction of CsV_3Sb_5, and this pattern is the same as that shown in Supplementary Fig. 4c. **b**,**d**, Theoretically simulated Laue diffraction pattern along 001 direction, and this pattern is the same as that shown in Supplementary Fig. 4d. The detailed analyses of the characteristic spots in these patterns can be seen in Supplementary Note 2.

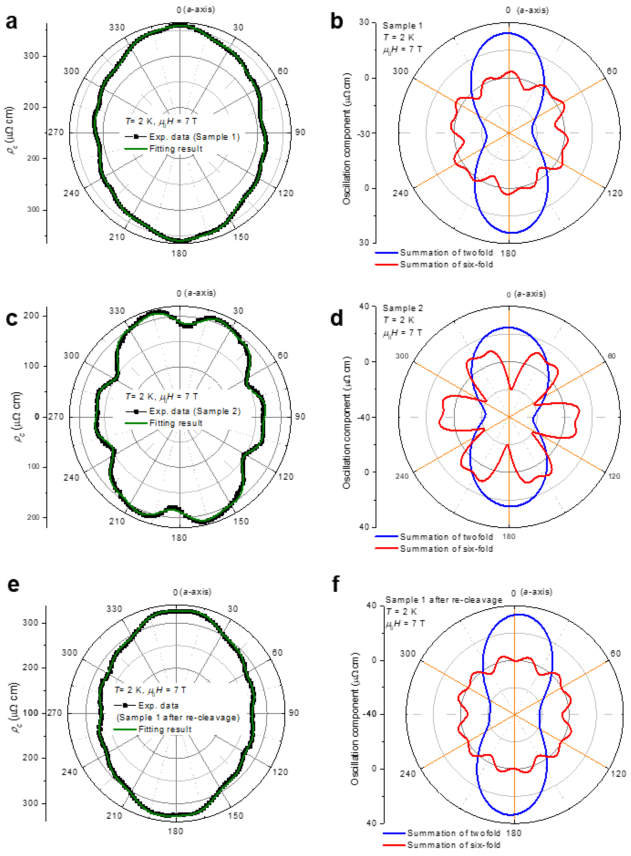

Supplementary Fig. 6 | Analysis results of twofold and six-fold components in $\rho_c(\theta)$ curves (T = 2 K, μ_0H = 7 T) measured in different samples. a,c,e, Fitting results to the experimental data measured in sample 1, sample 2, and re-cleaved sample 1, respectively. The formula for the fitting curve is 331.85 + 20.91cos[2(θ+ 2.42º)] + 3.39cos[4(θ+ 0.75º)] + 2.81cos[6(θ+ 7.97º)]+2.28 cos[12(θ– 3.79º)] μΩ·cm for sample 1; that is 180.61 + 23.50cos[2(θ– 1.36º)] + 1.6 8cos[4(θ+ 12.30º)] – 13.84cos[6(θ–3.73º)] – 6.10cos[12(θ– 34.29º)] μΩ·cm for sample 2; that is 293.70 + 27.70cos[2(θ– 3.18º)] + 6.05cos[4(θ–3.17º)] +2.43cos[6(θ+0.08º)] – 2.92cos[12(θ– 1.10º)] μΩ·cm for re-cleaved sample 1. **b,d,f**, Resistivity components from nematic and six-fold electronic states.

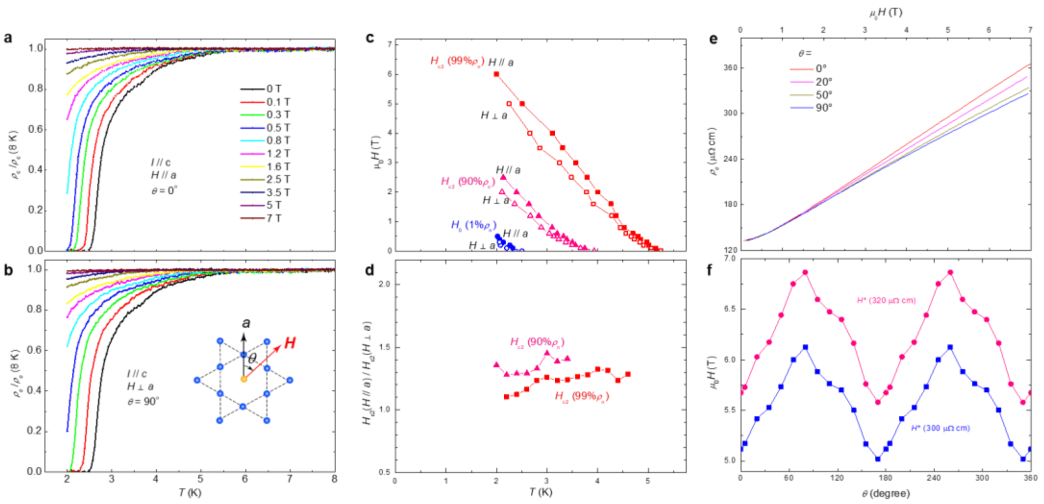

Supplementary Fig. 7 | In-plane anisotropy of the upper critical field measured in sample 1. a,**b**, Temperature dependent normalized *c*-axis resistivity measured under different magnetic field parallel and perpendicular to the *a*-axis, respectively. **c**, Temperature dependent upper critical field ($\mu_0 H_{c2}$) and zero-resistance field ($\mu_0 H_0$) obtained by using different criterions of *c*-axis resistivity. **d**, Temperature dependence of in-plane anisotropy of upper critical field. The averaged value is 1.35 ± 0.06 for the criterions of 90%ρ_n. **e**, Magnetic field dependence of *c*-axis resistivity measured with different angles between field and the *a*-axis at T = 10 K. **f**, Characteristic fields $\mu_0 H^*$ with the criterions of *c*-axis resistivity.

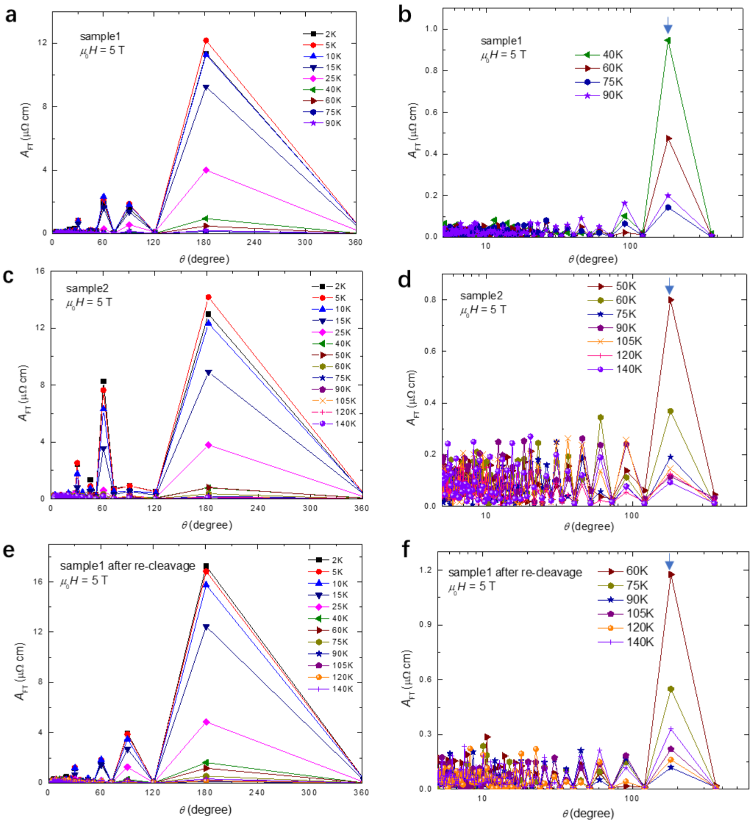

Supplementary Fig. 8 | Fourier transformation results to $\rho_c(\theta)$ curves measured at different temperatures under 5 T. a,**c**,**e**, FT results obtained in sample 1, sample 2, and re-cleaved sample 1, respectively. **b**,**d**,**f**, The semilog plots of FT results obtained at high temperatures. One can see that the amplitude of the 180º (or C_2) peak is reduced to the background signal when the temperature is increased to about 90 K, and the amplitude is always in the noise level at high temperatures above T_{CDW}.

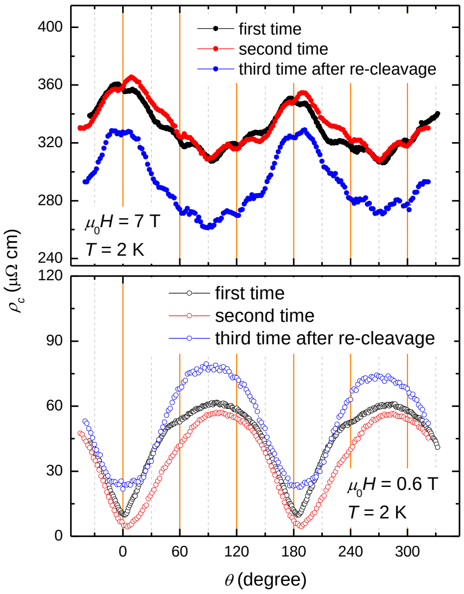

Supplementary Fig. 9 | Repeatability of *c*-axis resistivity measurements in the same sample (Sample 1). Raw data of $\rho_c(\theta)$ measured by two rounds of experiments with the same electrodes (black and red symbols). Before the second round of measurements, the sample was rotated with an in-plane angle of about 10 degrees on the sample holder compared with the first round of measurements. The parallelism of the *ab*-plane and the magnetic field is also adjusted a bit in the second round. However, the twofold nature of $\rho_c(\theta)$ curves are highly repeatable when considering the initial angle difference. We also re-cleave sample 1 and re-measure the *c*-axis resistivity, and the experimental data are shown as blue symbols in the figure. The calculated resistivity is slightly changed in the re-cleaved sample, but the twofold nature of $\rho_c(\theta)$ curves are also repeatable.

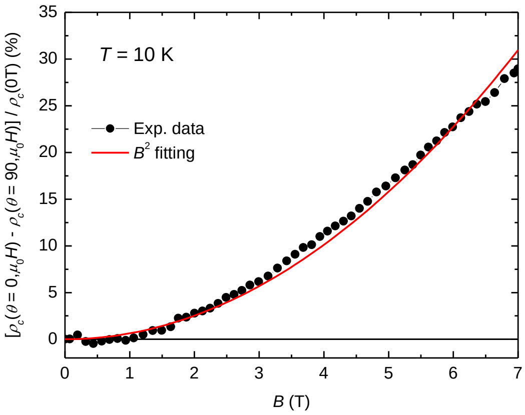

Supplementary Fig. 10 | Magnetic field dependence of resistivity difference measured in sample 1. The experimental data of $\rho_c(\theta = 90^{\circ}) - \rho_c(\theta = 0^{\circ})$ are derived from Supplementary Fig. 7e. The solid line is the fitting result by using a quadratic term of the magnetic field. One can see that the resistivity difference is roughly proportional to B^2. The result suggests that the twofold nature emerges at a very small field and is enlarged by a high magnetic field.

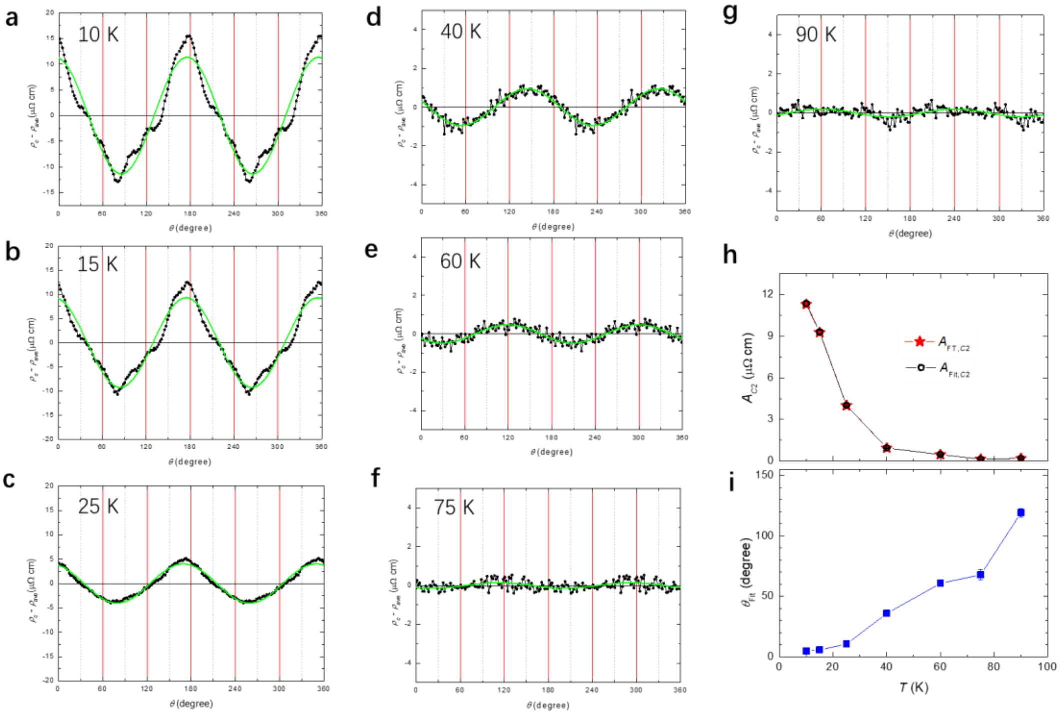

Supplementary Fig. 11 | Angular dependence of *c*-axis resistivity measured at different temperatures in sample 1. a-g, Angular dependence of *c*-axis resistivity measured at different temperatures under 5 T, and the solid lines are the fitting results by cosine functions with the periods of 180º. One can see that the twofold feature can be observed in the curve measured at 75 K, but the feature is almost disappeared at 90 K which is near T_{CDW}. However, the six-fold feature cannot be clearly identified at about 40 K. **h**, Temperature dependence of the amplitude of the twofold component. $A_{Fit,C2}$ is the fitting parameter of the cosine functions shown in **a-g**, and $A_{FT,C2}$ is the C_2 amplitude in the FT results shown in Supplementary Fig. 8a. One can see the consistence of the two sets of data. **i**, Temperature dependence of phase of the cosine functions in **a-g**. One can see an obvious phase shift with increase of temperature, which suggests that the contribution from other domains may increase. The error bars in **h** and **i** are determined from the fitting procedure.

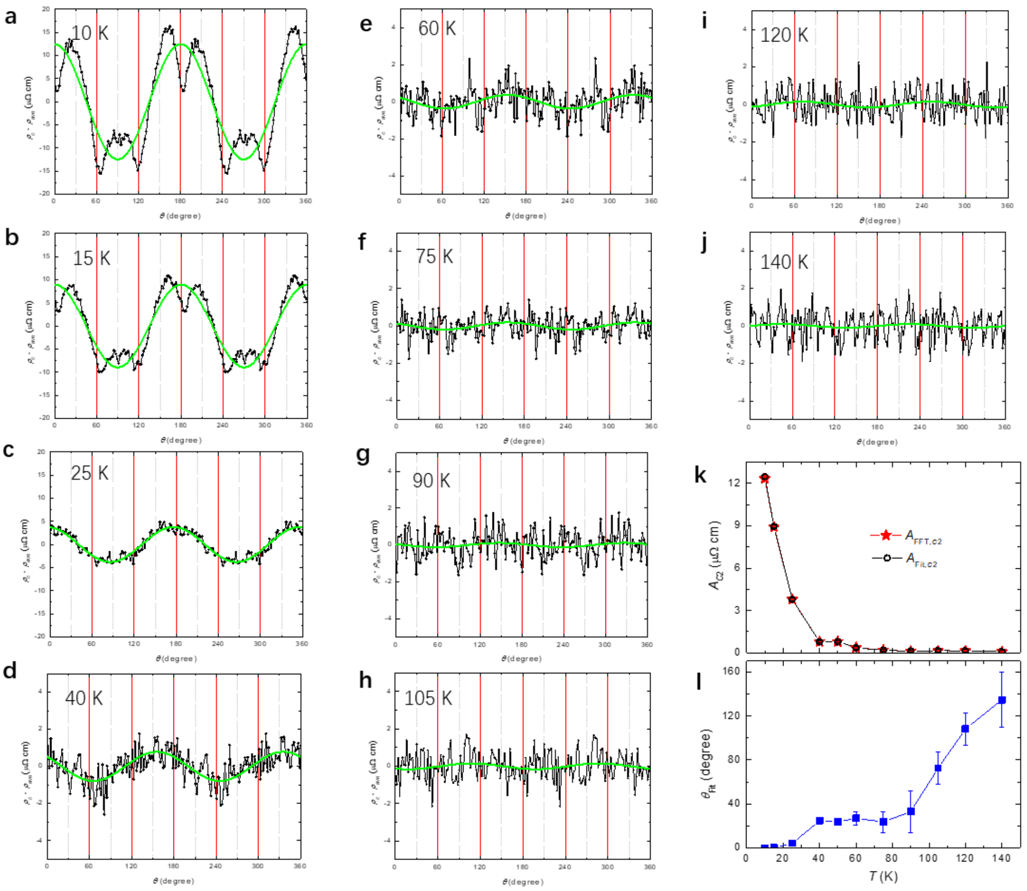

Supplementary Fig. 12 | Angular dependence of *c*-axis resistivity measured at different temperatures in sample 2. a-j, Angular dependence of *c*-axis resistivity measured at different temperatures under 5 T, and the solid lines are the fitting results by cosine functions with the periods of 180º. One can see that the twofold feature can be observed in the curve measured at 75 K, but the feature is almost disappeared at 90 K which is near T_{CDW}. However, the six-fold feature cannot be clearly identified at about 40 K. **k**, Temperature dependence of the amplitude of the twofold component. $A_{\mathrm{Fit},C2}$ is the fitting parameter of the cosine functions shown in **a-j**, and $A_{\mathrm{FT},C2}$ is the C_2 amplitude in the FT results shown in Supplementary Fig. 8c. One can see the consistence of the two sets of data. **l**, Temperature dependence of the phase of the cosine functions in **a-j**. One can see an obvious phase shift with increase of temperature, which suggests that the contribution from other domains may increase. The error bars in **k** and **l** are determined from the fitting procedure.

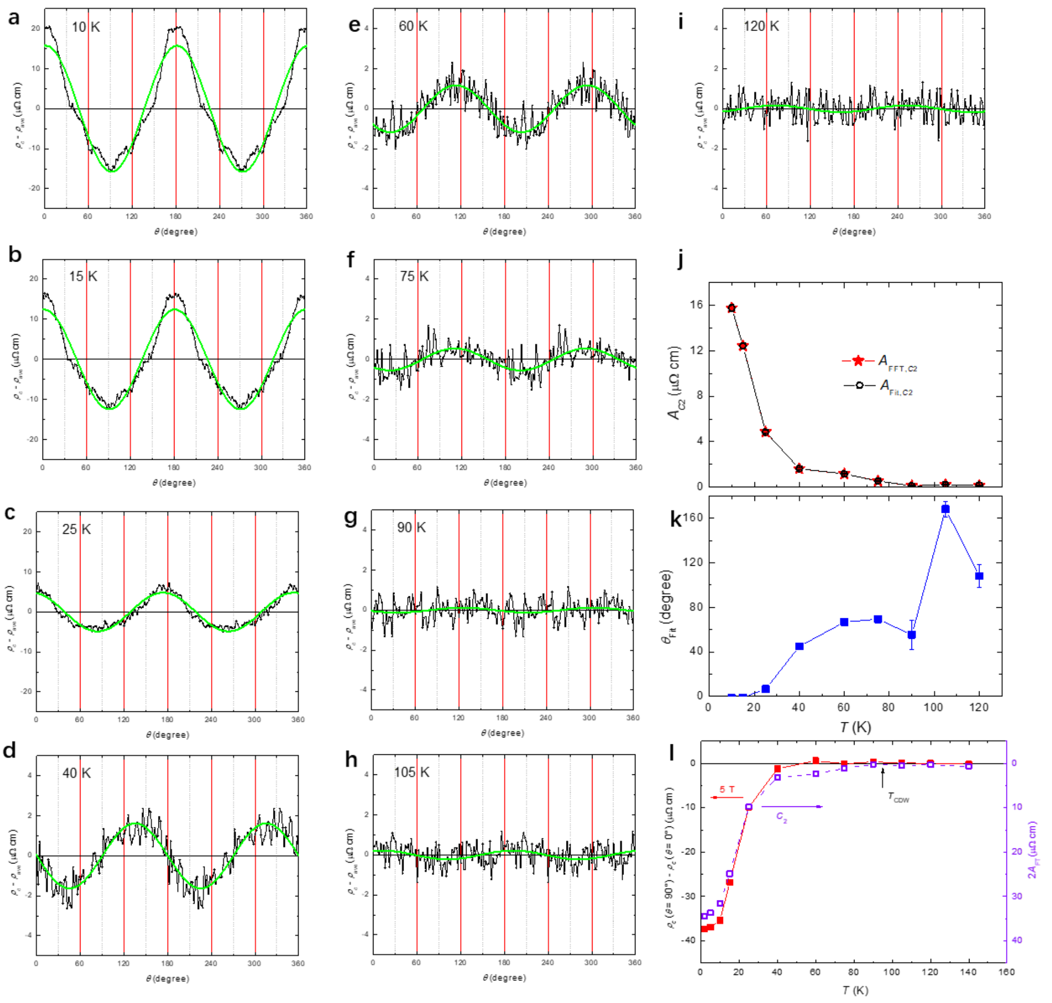

Supplementary Fig. 13 | Angular dependence of *c*-axis resistivity measured at different temperatures in re-cleaved sample 1. a-i, Angular dependence of *c*-axis resistivity measured at different temperatures under 5 T, and the solid lines are the fitting results by cosine functions with the periods of 180º. One can see that the twofold feature can be observed in the curve measured at 75 K, but the feature is almost disappeared at 90 K which is near T_{CDW}. However, the six-fold feature cannot be clearly identified at about 40 K. **j**, Temperature dependence of the amplitude of the twofold component. $A_{Fit,C2}$ is the fitting parameter of the cosine functions shown in **a-i**, and $A_{FT,C2}$ is the C_2 amplitude in the FT results shown in Supplementary Fig. 8e. One can see the consistence of the two sets of data. **k**, Temperature dependence of the phase of

the cosine functions in **a**-**i**. One can see an obvious phase shift with increase of temperature, which suggests that the contribution from other domains may increase. The error bars in **j** and **k** are determined from the fitting procedure. **l**, Temperature dependence of the normal-state nematicity. The solid symbols are derived from the *c*-axis resistivity difference between $\theta = 0^{\circ}$ and 90° at 5 T, while the open symbols show twice the amplitude of the 180° (or C_2) peak ($2A_{\mathrm{FT},C2}$) based on **j**.

Supplementary References

[1] Li, H. et al. No indication of chiral flux current in the topological kagome metal CsV_3Sb_5. Preprint at http://arxiv.org/abs/2107.11326 (2021).
